# Alterations in cathepsin H activity and protein patterns in human colorectal carcinomas

**DOI:** 10.1054/bjoc.1999.1098

**Published:** 2000-03-06

**Authors:** E C del Re, S Shuja, J Cai, M J Murnane

**Affiliations:** Department of Pathology, Mallory Institute of Pathology, Department of Biochemistry, Boston University School of Medicine, L804, 715 Albany Street, Boston, MA, 02118, USA

**Keywords:** cathepsin H, cysteine proteinases, colorectal cancer

## Abstract

Our analyses of cathepsin H activity levels and protein forms in human colorectal cancers compared to matched control mucosa support the concept that altered proteinase expression patterns may reflect both cancer stage and site. Cathepsin H-specific activity was significantly increased in colorectal cancers compared to control mucosa (*P* = 0.003;*n* = 77). Highest specific activities and cancer/normal ratios (C/N) for activity were measured in Dukes' B and C stage carcinomas, cancers involved in local spread and invasion to lymph nodes. In contrast, cathepsin B and L activities analysed in the same paired extracts had been shown to be most frequently elevated in earlier stage carcinomas (Dukes' A and B), confirming that cathepsin H demonstrates a distinct pattern of expression during colorectal cancer progression. Although cathepsin H activities were most commonly elevated in Dukes' C cancers at all colon sites, both specific activity and C/N ratios were significantly higher for cancers of the left colon compared to other colon locations. A subset of 43 paired extracts analysed on Western blots also revealed consistent changes in cathepsin H protein forms in cancers. Normal mucosa typically showed a strong protein doublet at 31 and 29 kDa while cancers demonstrated decreased expression or total loss of the 31 kDa protein (90% of cases), equal or increased expression of the 29-kDa protein (67% of cases) and the new appearance or up-regulation of a cathepsin H band at 22 kDa (78% of cases). C/N ratios for cathepsin H enzyme activity correlated significantly with C/N ratios for the 29 kDa mature single-chain protein form (*P*< 0.001), with increased activity most commonly associated with elevated expression of 29-kDa cathepsin H but also with up-regulation of the 22-kDa band, suggesting a shift to more fully processed, mature active cathepsin H protein forms in cancers. Changes in cathepsin H expression were also detected by immunohistochemistry as elevated cathepsin H staining in tumour epithelial cells. © 2000 Cancer Research Campaign


*British Journal of Cancer* (2000) **82**(7), 1317–1326
© 2000 Cancer Research Campaign
DOI: 10.1054/ bjoc.1999.1098, available online at http://www.idealibrary.com on IDEAL®

### Alterations in cathepsin H activity and protein patterns in human colorectal carcinomas

**EC del Re[1], S Shuja[1,2], J Cai[1] and MJ Murnane[1,2,3]**

[1]Department of Pathology, [2]Mallory Institute of Pathology, [3]Department of Biochemistry, Boston University School of Medicine, L804, 715 Albany Street, Boston, MA 02118, USA

**Summary** Our analyses of cathepsin H activity levels and protein forms in human colorectal cancers compared to matched control mucosa support the concept that altered proteinase expression patterns may reflect both cancer stage and site. Cathepsin H-specific activity was significantly increased in colorectal cancers compared to control mucosa ($P = 0.003$; $n = 77$). Highest specific activities and cancer/normal ratios (C/N) for activity were measured in Dukes' B and C stage carcinomas, cancers involved in local spread and invasion to lymph nodes. In contrast, cathepsin B and L activities analysed in the same paired extracts had been shown to be most frequently elevated in earlier stage carcinomas (Dukes' A and B), confirming that cathepsin H demonstrates a distinct pattern of expression during colorectal cancer progression. Although cathepsin H activities were most commonly elevated in Dukes' C cancers at all colon sites, both specific activity and C/N ratios were significantly higher for cancers of the left colon compared to other colon locations. A subset of 43 paired extracts analysed on Western blots also revealed consistent changes in cathepsin H protein forms in cancers. Normal mucosa typically showed a strong protein doublet at 31 and 29 kDa while cancers demonstrated decreased expression or total loss of the 31 kDa protein (90% of cases), equal or increased expression of the 29-kDa protein (67% of cases) and the new appearance or up-regulation of a cathepsin H band at 22 kDa (78% of cases). C/N ratios for cathepsin H enzyme activity correlated significantly with C/N ratios for the 29 kDa mature single-chain protein form ($P < 0.001$), with increased activity most commonly associated with elevated expression of 29-kDa cathepsin H but also with up-regulation of the 22-kDa band, suggesting a shift to more fully processed, mature active cathepsin H protein forms in cancers. Changes in cathepsin H expression were also detected by immunohistochemistry as elevated cathepsin H staining in tumour epithelial cells. © 2000 Cancer Research Campaign

**Keywords:** cathepsin H; cysteine proteinases; colorectal cancer

Cathepsin H is a lysosomal glycoprotein and a member of the cysteine proteinase family, as are cathepsins B and L (Kirschke et al, 1998). These proteinases have been viewed traditionally as simple mediators of lysosomal protein degradation (Chapman et al, 1997), although studies have also demonstrated their temporal regulation during development (Mathur et al, 1997) and their specificity of expression with cell type and function (Taniguchi et al, 1993). Cathepsin H is easily distinguished from cathepsins B and L by its unique aminopeptidase activity (Guncar et al, 1998) while the latter are better described as endopeptidases (Kirschke et al, 1998).

Cathepsins have been particularly well described for their abnormal expression patterns during cancer development (Iacobuzio-Donahue et al, 1997; Leto et al, 1997). Cancer growth and progression require proteolytic enzymes as necessary tools for invasion and metastasis (Liotta and Stetler-Stevenson, 1991). Recent findings suggest that various types of cysteine proteinase have specific roles in apoptosis (Nitatori et al, 1996), the cell cycle (Fu et al, 1998), MHC class II immune responses (Lafuse et al, 1995) or activation of growth factors and hormones (Mithofer et al, 1998). Matrix metalloproteinases, serine and aspartic proteinases, as well as cysteine proteinases, contribute to the degradation of basement membranes and the extracellular matrix (Gabrijelcic et al, 1992) during cancer progression. Evidence has now accumulated that these proteinases may also serve as prognostic indicators in solid cancers (Schwartz, 1995).

*Received 3 November 1998*
*Revised 12 August 1999*
*Accepted 7 September 1999*

*Correspondence to:* MJ Murnane

While fewer studies on cathepsin H in cancer have been done compared to other cathepsins, cathepsin H protein has been found to be significantly elevated in sera of breast cancer patients and in breast cancer tissue extracts (Gabrijelcic et al, 1992). Cathepsin H has also been detected by immunohistochemistry in primary melanoma and metastatic lesions compared to pigmented naevi or normal skin (Kageshita et al, 1995). It has been found to be secreted, in its precursor form, from the human melanocytic cell line, G361, into culture media (Tsushima et al, 1991). Elevated cathepsin H serum levels, measured by enzyme-linked immunosorbent assay (ELISA) in patients with melanomas correlated with the presence of metastases and with decreased patient survival (Kos et al, 1997). In patients with gliomas, elevated cathepsin H also correlated with increasing malignant potential of the cancers (Sivaparvathi et al, 1996).

In contrast, decreased cathepsin H expression has been reported in some cancers. For example, cathepsin H, detected by ELISA, in squamous cell carcinomas of the head and neck, was found to be higher in normal compared to malignant cell extracts (Kos et al, 1995), with high normal cathepsin H concentrations correlating with longer disease-free survival (Budinha et al, 1996). Cathepsin H enzyme activity was also diminished in polycystic kidney disease, a non-cancer condition, characterized by hyperproliferation (Hartz and Wilson, 1997). Still other studies have measured

**Table 1** Patient characteristics

| Characteristic | *n*[a] |
|---|---|
| Patients | 77 |
| Male/female | 49/27 |
| Median age in years (range) | 70 (36–86) |
| Stage | |
| A | 12 |
| B | 30 |
| C | 20 |
| D | 15 |
| Tumour location | |
| Right | 38 |
| Left | 36 |

[a]Subtotals do not always add up to the total number of patients studied, because of incomplete patient information for some cases.

higher cathepsin H gene expression in well differentiated pancreatic cancer cells compared to less differentiated cancer cells (Paciucci et al, 1996). These studies suggest a complex association between cathepsin H and altered cell growth patterns.

Reported differences in cathepsin H expression patterns in various cancers may reflect highly specific functions for cathepsin H in different tissues and at different cancer stages. Our current studies of cathepsin H-specific aminopeptidase activity, cathepsin H protein forms and immunohistochemistry in matched pairs of colorectal cancers and control mucosae, were designed to explore further the specificity of this proteinase with respect to cancer stage and site in colorectal cancer progression.

### MATERIALS AND METHODS

### Tissue specimens

Tissues were collected at surgery as colectomy specimens at the Mallory Institute of Pathology or through the Cooperative Human Tissue Network (CHTN), with cancer samples and normal mucosa obtained from each patient. Matched normal mucosa, resected at a minimum of 5–10 cm from the cancer tissue, was used as the control tissue as colorectal cancers originate from the epithelium of the colon mucosa. Care was taken to eliminate necrotic portions from the cancers. In addition, fat, serosa and muscle layers were separated from the normal mucosa epithelium prior to freezing. All tissues collected were stored frozen at – 80°C until needed for experiments.

### Patients

Seventy-seven colorectal cancer patients were included in our cathepsin H expression study. Table 1 summarizes information on patient characteristics.

### Dukes' classification

Before reaching the laboratory, cancer tissues were staged by pathologists at the Mallory Institute of Pathology, or at CHTN-associated hospitals, according to the Dukes' classification system (Dukes, 1932) as modified by Turnbull et al (1967). Dukes' A cancers are defined as those confined to the bowel wall, Dukes' B cancers as those spread through the wall without involvement of lymph nodes, Dukes' C cancers as having metastasized to the regional lymph nodes regardless of the extent of bowel wall penetration, and Dukes' D cancers as having metastasized to distant sites.

### Tissue extractions

Tissue extractions were carried out concomitantly for each cancer tissue and matched normal mucosa, as previously described (Iacobuzio-Donahue et al, 1997). Tissue samples (60–80 mg in 400–500 μl double-distilled deionized water) were homogenized using a Polytron homogenizer. The homogenized tissues were sequentially frozen three times in a methanol/dry ice bath (approximately –79°C) and thawed three times in warm water (approximately 37°C), in order to rupture the cells and release their contents. Centrifugation was carried out for 50 min at 4°C at 12 000 rpm (17 210 ***g***) in a Sorvall 5B centrifuge. The supernatants, containing extracted soluble protein, were removed and stored at – 80°C until needed. In order to control for possible alterations of cathepsin H protein during the extraction procedure, the case no. 64 cancer sample was extracted independently either in the presence of four different inhibitors or, using standard procedure, in water (dd$H_2O$). The inhibitors were E-64 (100 μM, L-trans-epoxysuccinylleucylamido(4-guanidine)butane (Sigma, St Louis, MO, USA); phenylmethylsulphonyl fluoride (PMSF; 200 μM) (Sigma, St Louis, MO, USA); EDTA (10 mM) or pepstatin (1 mg ml$^{-1}$) (Sigma). All five extraction samples were then compared for variations in cathepsin H banding patterns on Western blots.

### Cathepsin H enzyme activity assay

The activity of cathepsin H was determined against the specific synthetic substrate L-Arg-MNA (Sigma; and Enzyme System Products, Dublin, CA, USA) to take advantage of the specific aminopeptidase activity of cathepsin H, that distinguishes it from other cysteine lysosomal enzymes, such as cathepsins B and L (Schwartz and Barrett, 1980; Guncar et al, 1998). Tissue extracts (10–20 μl per assay) were pre-incubated in assay buffer (0.1 M MES-EDTA buffer, 1 mM dithiothreitol (DTT), pH 6.8) at 37°C for 5 min, using a modification of methods described by Schwartz and Barrett (1980). To start the assay reaction, 1 mM substrate (L-Arg-MNA) was added to buffer plus tissue extract, samples incubated at 37°C for 10 min, and then reaction stopped by addition of 50 μl 1 N hydrochloric acid (HCl) in 2% Triton X-100. Fast blue (*O*-dianisidine tetrazotized) (Sigma) (0.5 mg ml$^{-1}$) was added and colour developed for 10 min before absorbance was read at 520 nm in a Gilford spectrophotometer. Protein content (mg ml$^{-1}$) was determined for each tissue extract by the method of Lowry et al (1951), using bovine serum albumin as standard. Enzyme activity in nmol of substrate hydrolysed per min ml$^{-1}$ was always normalized for protein content, with final specific activity defined as nmol of substrate hydrolysed per min mg$^{-1}$ of protein (nmol min$^{-1}$ mg$^{-1}$). Cathepsin H was also tested in the presence and absence of the inhibitor puromycin at both 1 mM (Schwartz and Barrett, 1980) and 10 μM concentrations (Claus et al, 1998), to control for the specificity of the activity measured in tissue homogenates (Kirschke et al, 1998). Cathepsin H activity was also measured in the presence and absence of E-64 (L-trans-epoxysuccinyl-leucylamido(4-guanidino)butane) (100 μM in dimethyl sulphoxide) as described by Barrett et al (1982).

 © 2000 Cancer Research Campaign

### Western blotting assay

Colorectal cancer and matched mucosa extracts (30 μg sample protein per lane) were added to a denaturing buffer (2% sodium dodecyl sulphate (SDS), 5% β-mercaptoethanol, 10% glycerol, 62.5 mM Tris, pH 6.8) and boiled for 5 min. The samples were then run on a 16% polyacrylamide minigel (Novex, San Diego, CA, USA) for 3.5 h at 70 V on a Biorad Mighty Small 260 apparatus. Rainbow size markers (Amersham, Arlington Heights, IL, USA) were run on each gel (5 μl per slot) as MW standards. After electrophoresis the gel proteins were transferred overnight to a nitrocellulose membrane (Schleicher and Shuell, Keene, NH, USA) in a Biorad Transblot Apparatus at 100 mAmp (Towbin et al, 1979), containing transfer buffer (25 mM Tris, 192 mM glycine, 20% methanol, 1% SDS). After transfer, the membrane was washed in TBST buffer (0.25 mM Tris, 125 mM sodium chloride (NaCl), 0.05% Tween-20), and then blocked with 5% milk (dry milk) in TBST, before incubating with cathepsin H primary antibody overnight (Athens Research and Technology, Athens, GA, USA) that had been diluted 1:2000 in 5% milk in TBST. Blots were washed again with 5% milk in TBST and then with TBST before incubation with horseradish peroxidase conjugated secondary antibody (Sigma) for 1 h. Blots were then washed for 3 h in 5% milk in TBST and then three times for 10 min in TBST. Proteins were detected by using an enhanced chemiluminescent system (NEN, Boston, MA, USA).

### Laser densitometry

Relative quantities of cathepsin H protein forms in 43 paired extracts detected on film, following Western blotting, were visually assessed by three independent observers and a C/N ratio determined for each case. Confirmation of these C/N ratios were then determined for 34 cases by quantitative laser densitometry, using a Molecular Dynamics Personal Densitometer SI.

### Immunohistochemistry

Archival paraffin-embedded tissue sections of colorectal carcinomas were obtained from the Mallory Institute of Pathology. Tissue sections (5 microns) were sliced using a microtome, mounted on poly-lysine-coated clean glass slides, dewaxed in xylene and hydrated in graded concentrations of ethanol. Endogenous peroxidase was blocked with 0.3% hydrogen peroxide in methanol after which slides were blocked 5 min with a 1:20 dilution of casein. Next, slides were incubated with a 1:2500 dilution of polyclonal rabbit anti-human liver cathepsin H antibody (Athens Research and Technology) diluted in 0.05 M Tris buffer, pH 7.4, for 30 min at room temperature, followed by a 5 min incubation at 37°C with biotinylated secondary antibody (BioGenex), then a 5-min incubation at 37°C with peroxidase-conjugated streptavidin label (BioGenex). Slides were washed three times with 0.05 M Tris buffer, pH 7.4, after each incubation. Colour was developed using DAB (3-3′ diaminobenzidine tetrahydrochloride). Haematoxylin was used to counterstain the slides. For control slides, the primary antibody was replaced by 0.05 M Tris buffer, pH 7.4. Positive or negative expression and subcellular localization of cathepsin H protein in tissue sections was evaluated by two independent observers. Intensity was scored on a scale of 0–4, with 0 equal to negative expression and 4 equal to strongly positive expression.

### Statistical analysis

Summary data are expressed as the mean ± standard deviation (s.d.) unless otherwise indicated. To compare data with two means, the Student's *t*-test for the difference between means was used. To test whether a population mean was different from 1, a one-sample *t*-test was used (Dawson-Saunders and Trapp, 1994). To test for the association between two variables, linear regressions with calculation of a correlation coefficient have been used. To compare differences between distributions, the $\chi^2$ test has been used with a Fisher exact test correction where needed. To compare medians of two groups, the Wilcoxon rank sum test was used. Confidence limits were also calculated in order to determine the mean difference in our paired study between cancers and control mucosae activities, by subtracting the cathepsin H-specific activity for each control mucosa from the cathepsin H-specific activity for each matched cancer tissue, and then calculating a mean difference for these multiple individual calculations and a mean standard error of the difference, according to the standard formula $d \pm (t\ value)(SE_d)$ = confidence interval (Dawson-Saunders and Trapp, 1994).

Two-tailed probability values of 0.05 or less were considered significant, unless a one-tailed probability test is indicated. Calculations were performed using the statistical package, GraphPad InStat (GraphPad Software, version 1.11a, C Haudenschield, 1990) and confirmed by use of Microsoft Excel 5.0 (Microsoft Corp) or 'Primer of Biostatistics', Version 4.0 (McGraw-Hill, 1996).

### RESULTS

Our analyses of cathepsin H aminopeptidase activity and protein banding patterns were carried out on matched pairs of colorectal cancer and control mucosa tissue samples. The 77 cases analysed included 12 Dukes' A cancers, 30 Dukes' B cancers, 20 Dukes' C cancers and 15 Dukes' D cancers (Table 1), each with patient-matched normal mucosa.

### Cathepsin H mean aminopeptidase activity and mean C/N ratio

Cathepsin H demonstrated a mean specific aminopeptidase activity of $8.46 \pm 5.2$ nmol $min^{-1}$ $mg^{-1}$ in 77 normal mucosa tissues and an activity of $10.9 \pm 8.3$ nmol $min^{-1}$ $mg^{-1}$ in the 77 matched colorectal cancer specimens from the same patients. This difference in cathepsin H activity between cancer tissues and control mucosa was highly significant ($P = 0.003$) (Figure 1A). Calculation of the 95% confidence interval for the mean increase in cathepsin H enzyme-specific activity for 77 pairs of cancer and matched normal mucosa indicated a range of values between 1.3 nmol $min^{-1}$ $mg^{-1}$ and 3.76 nmol $min^{-1}$ $mg^{-1}$, an interval that does not include a zero value, confirming a significant increase in cathepsin H enzyme activity in colorectal cancers versus normal mucosa.

Cathepsin H specific activity in each cancer was also compared to the matched mucosa to determine an individual C/N ratio for each matched tissue pair. The mean cathepsin H C/N ratio for all 77 cases analysed was $1.43 \pm 0.77$ ($P < 0.0001$), indicating that cathepsin H activity was, on average, 1.4-fold higher in cancers than in matched normal mucosa (Figure 1B).

© 2000 Cancer Research Campaign

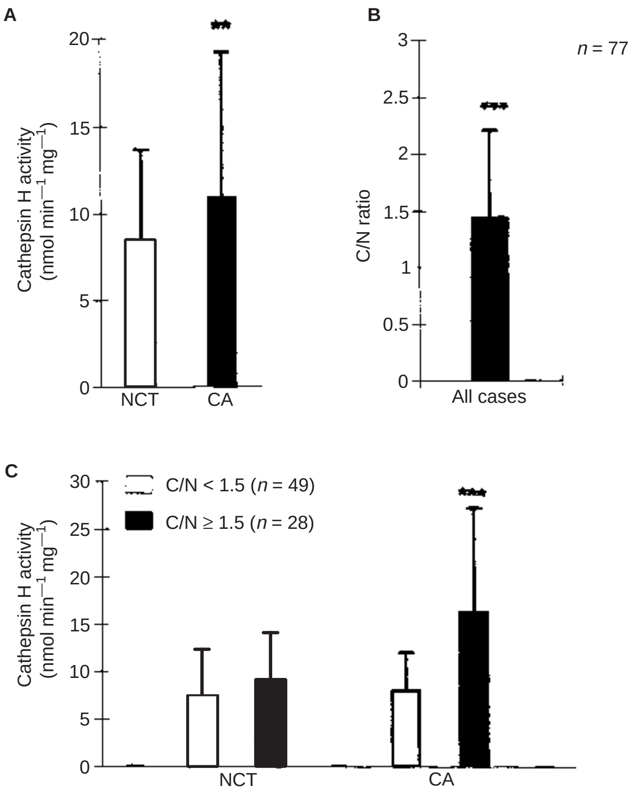

**Figure 1** Cathepsin H enzyme-specific activity and C/N ratios in colorectal cancer. (**A**) Cathepsin H enzyme specific activity was significantly elevated in colorectal cancer compared to matched normal colon mucosa ($n = 77$; $P = 0.003$, paired *t*-test) (**B**) The mean C/N ratio for 77 matched cancer and normal colorectal cases was found to be significantly different from 1 ($n = 77$, one-sample *t*-test). (**C**) Mean cathepsin H activity levels are graphed for normal mucosa from either 'low' or 'high' C/N groups compared to mean activity levels in cancers from 'low' or 'high' C/N groups. Cathepsin H activity levels in cancers with C/N ratios ≥ 1.5 were significantly elevated over mean activity levels for any other group. ***$P < 0.0001$. NCT, normal colon mucosa; CA, colorectal carcinoma

### Cathepsin H: frequency of high C/N ratios

As a further means of assessing the changes in cathepsin H aminopeptidase activity in cancers compared to matched normal mucosa, we ascertained the percentage of cases that demonstrated a 'high' C/N ratio, defined as C/N ≥ 1.5, thus including only those cases having a C/N ratio above the mean increase for all cancers assayed (mean C/N = 1.4; $P = 0.0001$). By definition such cancers have an increase in activity at least 50% over normal, or the equivalent of an additional cathepsin H gene. Of 77 matched pairs analysed, 28 cases (36%) demonstrated a C/N ratio ≥ 1.5. The mean C/N ratio for these 28 cases was 2.22 ± 0.7, while the mean C/N ratio for the remaining 49 cases (64%) was 0.98 ± 0.26 ($P = 0.0001$), suggesting that the increase in cathepsin H activity in all cases could be explained primarily by elevated cathepsin H activity levels in 1/3 of the cancers analysed, while the remaining 2/3 of the cancers demonstrated little change in cathepsin H activity levels. We subsequently compared activity levels in the respective normal and cancer tissues for cases with C/N ratios ≥ 1.5 compared to cases with C/N < 1.5. Cathepsin H enzyme activity levels in normal mucosa specimens from patients with either 'low' or 'high' C/N ratios were not statistically different (7.5 ± 4.6 and 8.99 ± 5.46 nmol $min^{-1}$ $mg^{-1}$ respectively). These normal mucosa activity levels were also indistinguishable from cathepsin H activity levels in the cancers with 'low' C/N ratios (7.9 ± 3.9 nmol $min^{-1}$ $mg^{-1}$), as graphed in Figure 1C. However, cancers with 'high' C/N ratios demonstrated a mean cathepsin H enzyme activity of 16.14 ± 10.9 nmol $min^{-1}$ $mg^{-1}$, more than twice the activity in control tissues or in cancer tissues with 'low' C/N ratios ($P < 0.0001$). These results provide evidence for two populations of colorectal cancers: those demonstrating cathepsin H enzyme activity at least twice that of matched control mucosa and those demonstrating no change in cathepsin H enzyme activity compared to normal mucosa.

### Cathepsin H enzyme activity: controls for assay specificity

Proteinases able to hydrolyse single amino acid naphthylamide substrates in mammalian cells include the metalloexopeptidase arylamidases, a subclass of the cytosolic aminopeptidases (Johnson and Hersh, 1990) and the cysteine proteinase cathepsin H, the only known aminopeptidase of the mammalian lysosome (Kirschke et al, 1998). Specificity of the assay was demonstrated by measuring cathepsin H enzyme activity in the presence and absence of two different concentrations of the inhibitor puromycin (10 μM and 1 mM). At the 10 μM micromolar concentration, puromycin is a strong inhibitor of cytosolic aminopeptidases (Kirschke et al, 1998) but does not inhibit cathepsin H. However, at the 1 mM concentration, puromycin generates a partial inhibition of cathepsin H (Schwartz and Barrett, 1980). Assays of homogenates from six matched cancer and normal samples, two tumour metastases and one adenoma showed no differences in activity in the presence or absence of 10 μM puromycin, indicating that the assay detected cathepsin H enzyme activity and not the activity of cytosolic aminopeptidases. Assays of homogenates from eight matched cancer and normal samples showed partial inhibition of activity in the presence of 1 mM puromycin, with cancer tissues showing 22.7 ± 9.97% inhibition ($n = 8$) and matched normal tissues showing 26.0 ± 18.9% inhibition (not a significant difference). This partial inhibition by 1 mM puromycin was comparable to the 36% inhibition of purified cathepsin H by 1 mM puromycin reported in the literature (Schwartz and Barrett, 1980).

Cathepsin H enzyme activity was also measured in the presence and absence of 100 μM E-64 with no inhibition observed in either normal or cancer tissues, again demonstrating the specificity of our assay for cathepsin H activity. E-64 is a strong inhibitor of the majority of cysteine proteinases, with the exception of the cysteine proteinase cathepsin H, as reported in biochemical studies (Katunuma and Kominami, 1995) and more recently confirmed by the publication of the crystal structure of porcine cathepsin H (Guncar et al, 1998).

### Cathepsin H activity levels: correlation with Dukes' stage

To determine whether cathepsin H enzyme activity levels increased in a pattern related to cancer progression we calculated the mean enzyme-specific activity for cancers at each Dukes' stage and compared these activities to the mean specific activity for cathepsin H in control mucosa. As graphed in Figure 2A, cathepsin H demonstrated significantly elevated enzyme activity

 © 2000 Cancer Research Campaign

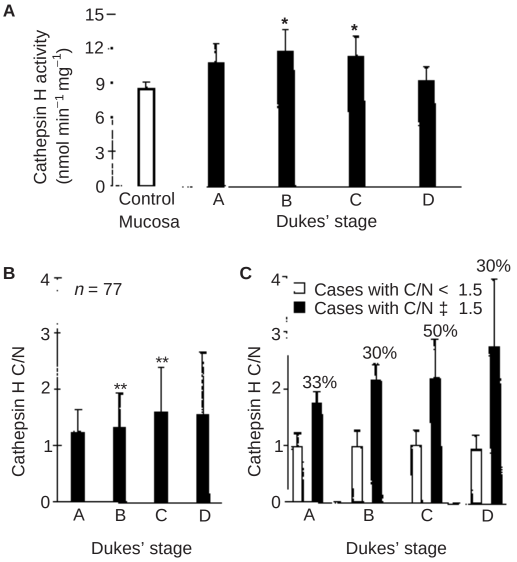

**Figure 2** Cathepsin H: changes with Dukes' stage. (**A**) Cathepsin H enzyme-specific activity (nmol min$^{-1}$ mg$^{-1}$ protein, mean ± s.e.m.), analysed by Dukes' stage, was significantly elevated in Dukes' B ($n$ = 30) and Dukes' C ($n$ = 20) cancers compared to cathepsin H enzyme-specific activity in control mucosa ($n$ = 77) (* $P < 0.05$, unpaired $t$-test, one-tailed value). (**B**) Cathepsin H C/N ratios (mean ± s.d.) analysed by Dukes' stages were 1.24 ± 0.41 for Dukes' stage A cancers ($n$ = 12), 1.33 ± 0.61 for Dukes' stage B cancers ($n$ = 30), 1.61 ± 0.80 for Dukes' stage C cancers ($n$ = 20) and 1.56 ± 1.11 for Dukes' stage D cancers ($n$ = 15). C/N ratios for Dukes' stage B and C cancers were significantly different from 1 (**$P < 0.01$). (**C**) C/N ratios (mean ± s.d.) for high and low C/N ratio cases with relative percentage shown of cases with high C/N ratios. For 77 cases analysed, 49 had C/N ratios < 1.5, with a mean C/N ≃ 1 (distribution by stage: A, $n$ = 8; B, $n$ = 21; C, $n$ = 10; D, $n$ = 10) while 28 cases had C/N ratios ≥ 1.5 (distribution by stage: A, $n$ = 4; B, $n$ = 9; C, $n$ = 10; D, $n$ = 5) with mean C/N ratios for these 'high' cases seemingly increasing with cancer progression. The highest percentage of cases with C/N ≥ 1.5 was in Dukes' stage C. For each Dukes' stage, 'high' C/N ratio cases had mean C/N ratios significantly higher than mean C/N ratios for 'low' C/N ratio cases ($P < 0.001$)

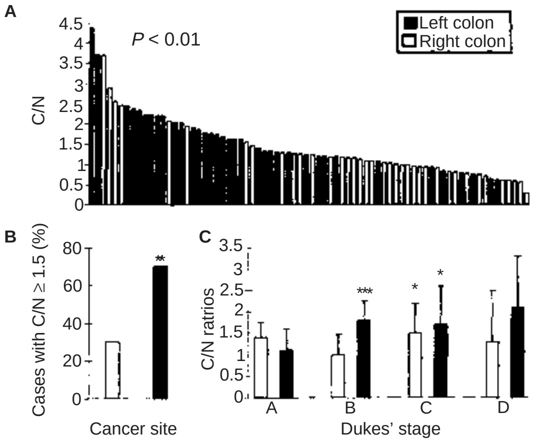

**Figure 3** Cathepsin H C/N ratios and tumour progression. (**A**) C/N ratios for 77 matched pairs of colorectal cancer and normal mucosa. Dark columns represent C/N ratios for left-sided cancers while light columns represent cancers from the right colon. Cancers located in the left colon had significantly higher C/N ratios than cancers from the right colon ($P < 0.01$) The mean C/N ratio for all right-sided or left-sided cancers were also significantly different from 1 ($P < 0.05$ and $P < 0.0001$ respectively). (**B**) The percentage of cases with C/N ratio ≥ 1.5 was significantly higher in left-sided cancers by the $\chi^2$ test ($P < 0.001$). (**C**) Mean cathepsin H C/N ratios in the left colon and in the right colon sorted by Dukes' stage. Dukes' C stage right-sided colon cancers had a mean C/N ratio significantly different from 1 (1.5 ± 0.7, $n$ = 10, *$P < 0.05$). Dukes' B and C left-sided colon cancers also demonstrated C/N ratios significantly different from 1 (1.8 ± 0.5, $n$ = 13, ***$P < 0.0001$ and 1.7 ± 0.7, $n$ = 10, *$P < 0.05$ respectively)

levels in Dukes' B and C stage cancers ($P$ = 0.02, $n$ = 30; and $P$ = 0.03, $n$ = 20 respectively; one-tailed, unpaired $t$-test) but not Dukes' A or D stage cancers compared to enzyme activity levels in normal mucosa.

To control for normal variation in cathepsin H activity levels in individual control mucosa, paired sample measurements were then utilized to derive average C/N ratios as an alternative measure of changes in cathepsin H activity with cancer stage (see Figure 2B). The C/N ratio for cathepsin H activity was most significantly elevated in stage B ($P$ = 0.006) and stage C cancers ($P$ = 0.003). Although Dukes' D cancers showed an average increase greater than that seen in Dukes' B cancers, these late stage cancers were also characterized by greater variability in cathepsin H expression levels, so that the overall increase was not as significant.

Finally, we also calculated the percentage of cases at each Dukes' stage that demonstrated a 'high' or 'low' C/N ratio as well as the average ratio at each stage for cathepsin H activity within the 'high' or 'low' expressing groups. These results demonstrated that the frequency of cases with C/N ≥ 1.5 in Dukes' C cases amounted to 50%, while in the other Dukes' stages it amounted roughly to 30% (Figure 2C). For all 'low' cases, irrespective of Dukes' stage, the mean C/N ratio was the same, that is ≃ 1.0. However, for cases in the 'high' C/N ratio group an apparent increase in mean C/N ratio was observed with cancer progression. Thus for Dukes' stage A cancers, the mean C/N ratio for cases with 'high' C/N ratios was 1.75 ± 0.19 ($n$ = 4) while the mean for 'high' C/N ratio cases for Dukes' stage B was significantly higher ($P < 0.01$, $n$ = 9, unpaired Student's $t$-test) and the mean C/N ratio for Dukes' stage D still higher (C/N = 2.76 ± 1.2, $n$ = 5), although the significance of this progressive elevation at stage D must be confirmed with more observations. For each Dukes' stage, cases in the 'high' group had significantly higher C/N ratios than cases in the 'low' C/N ratio group ($P < 0.001$).

### Cathepsin H activity levels: correlation with cancer location

In order to determine whether there was a correlation between cathepsin H activity and location of cancers in the large intestine, activity C/N ratios were analysed with respect to right-sided or left-sided cancer location, as shown in Figure 3A. We found that cancers resected from the left colon, including 19 sigmoid colon cancers, four rectosigmoid junction cancers and 11 rectal cancers, had a significantly higher mean C/N ratio (1.7 ± 0.8) than cancers resected from the right colon, including 23 caecum cancers, two ascending colon cancers and four transverse colon cancers ($P < 0.01$). However, although the C/N ratios were not as high in the right colon compared to the left colon, the mean C/N ratio for cathepsin H activity levels for all cases located in the right colon ($n$ = 38) was still significantly different from 1 (1.23 ± 0.69, $P < 0.05$).

To further assess the correlation between cathepsin H C/N ratios and cancer location, we also determined the percentage of cancers at each site that demonstrated a 'high' C/N ratio and found that among the cases with C/N ratios ≥ 1.5, 70% were resected from the left colon (Figure 3B, $P < 0.001$).

Significant differences in cathepsin H expression for left-sided versus right-sided cancers could be demonstrated not only by C/N

© 2000 Cancer Research Campaign

Cathepsin H C/N ratios sorted by cancer sites

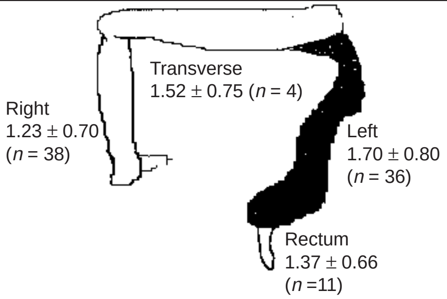

| Tumour locations | *n* | C/N ratios |
|---|---|---|
| Right colon | | |
| Caecum | 23 | 1.25 ± 0.77 |
| Ascending | 2 | 0.75 ± 0.28 |
| Transverse | 4 | 1.52 ± 0.75 |
| Total right | 38 | 1.23 ± 0.70* |
| Left colon | | |
| Sigmoid | 19 | 1.80 ± 0.78** |
| Rectosigmoid | 4 | 1.72 ± 0.47 |
| Rectum | 11 | 1.37 ± 0.66 |
| Total left | 36 | 1.70 ± 0.80*** |
| All sites | 74 | 1.45 ± 0.77*** |

**Figure 4** Cathepsin H activity C/N ratios sorted by colorectal cancer sites. Schematic drawing of the large intestine showing that cathepsin H C/N ratios were lowest in the right colon (light grey), increase in the transverse colon (medium grey) and were highest in the left colon (dark grey) Data were summarized for six specific regions of the right or left colon and C/N ratios shown for each region together with summary data. In addition to 34 left-sided cancers sorted by subgroups, the total of 36 left-sided cancers also included one cancer designated from the 'descending' colon and one cancer designated simply as 'left'. The 38 right-sided cancers included one 'hepatic flexure' cancer and eight cases simply termed 'right colon' cancers, as well as the 29 right-sided cancers sorted by regional subgroups. Mean C/N ratios for cathepsin H activity levels were significantly different from 1 for 38 colorectal cancers resected from the right colon ($P < 0.05$), 36 cancers from the left colon ($P < 0.0001$) and 19 cancers of the sigmoid colon ($P < 0.001$), as well as for the total of 74 colorectal cancers studied by tumour location ($P < 0.0001$)

ratios but also by a direct comparison of mean enzyme-specific activity levels for all cancers versus all normal mucosa at a given colon location. Cathepsin H activity for normal mucosa of the right colon (8.7 ± 5.2 nm min$^{-1}$ mg$^{-1}$ protein; $n = 38$) did not differ from activity for normal mucosa of the left colon (8.3 ± 5.3 nm min$^{-1}$ mg$^{-1}$ protein; $n = 36$). Cancers of the right colon also did not demonstrate a significant increase in activity (9.3 ± 6.6 nm min$^{-1}$ mg$^{-1}$ protein; $n = 38$; $P = 0.5$) over control mucosa but cancers of the left colon showed a highly significant increase in cathepsin H-specific activity (12.9 ± 9.7 nm min$^{-1}$ mg$^{-1}$ protein; $n = 36$) compared to control mucosa ($P = 0.0006$). The largest cancer subsets followed these patterns as well, with left-sided sigmoid cancers demonstrating a significant increase in cathepsin H activity (14.8 + 11.3 nm min$^{-1}$ mg$^{-1}$ protein; $n = 19$) over normal sigmoid mucosa (9.2 ± 6.5 nm min$^{-1}$ mg$^{-1}$ protein; $P = 0.006$) while the increase detected in right-sided caecal cancers (10.5 nm min$^{-1}$ mg$^{-1}$ protein; $n = 23$) versus normal caecal mucosa (9.0 ± 5.4 nm min$^{-1}$ mg$^{-1}$ protein; $P = 0.45$) was not significant.

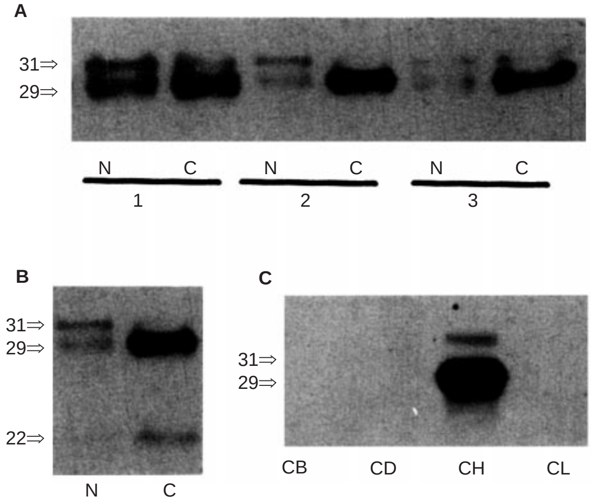

**Figure 5** Cathepsin H protein banding patterns: Western blots of matched cancer/normal pairs and purified cathepsin H protein controls. (**A**) Matched colorectal mucosa (N) and cancer (C) tissue extracts (30 μg total soluble protein/lane) from three independent cases demonstrated cathepsin H protein bands at 31 and 29 kDa. Decreased expression of the 31 kDa band was observed in the majority of colorectal cancers compared to the matched colon mucosa samples in conjunction with frequent increases in expression of the mature single-chain 29 kDa form of cathepsin H. (**B**) A representative matched pair of normal mucosa (N) and cancer (C) samples are shown, demonstrating the typical 31 plus 29 kDa bands in normal mucosa, decreased expression of the 31 kDa band in cancer plus the appearance of the 22 kDa heavy-chain of two-chain mature cathepsin H in the cancer, possibly reflecting increased cathepsin H protein processing in the cancer. (**C**) Four types of purified human liver cathepsins (100 ng per lane), including cathepsin B (CB), cathepsin D (CD), cathepsin H (CH) and cathepsin L (CL) were probed with the cathepsin H polyclonal antibody(Athens Research Laboratories, Athens, GA, USA) to confirm antibody specificity

We subsequently determined whether cathepsin H expression patterns for left-sided versus right-sided cancers would reflect the same specificity for Dukes' stage that had been observed for all cases. Among the 74 cancers studied for location effects, those resected from the left colon ($n = 38$) demonstrated similar distributions with respect to clinical stage to those from the right colon ($n = 36$). Thus, Dukes' C cancers from the right colon still demonstrated the highest C/N ratio (1.52 ± 0.7, $P < 0.05$), compared to all other right-sided cancers. In the left colon, both Dukes' stage B and C cancers demonstrated C/N ratios significantly different from 1 ($P < 0.0001$ and $P < 0.05$), as shown in Figure 3C. Thus, cancer stage appeared to predict cathepsin H activity C/N ratios, irrespective of site, but the combination of stage and site was the best predictor of elevated cathepsin H C/N ratios.

We have also used cathepsin H activity C/N ratios from every case to calculate a mean C/N ratio for cancers from each of six specific colorectal regions, as shown in Figure 4. While the mean C/N ratio for cancers resected from the transverse colon was found to be higher than the mean C/N ratio for cancers in the right colon, the number of cancers in the transverse colon was too small to determine whether the apparent stepwise increase in cathepsin H expression from right colon to transverse to left was significant (Figure 4).

### Cathepsin H protein expression patterns on Western blots

To confirm the results obtained by measuring cathepsin H activity in colorectal cancer compared to normal matched mucosa, cathepsin H protein banding patterns were also analysed by Western blotting studies of 43 paired extracts that had already been assayed for cathepsin H activity levels. The 43 cases included five Dukes' A stage cancers, 15 Dukes' B cancers, 13 Dukes' C cancers and 10 Dukes' D cancers.

In normal mucosa and in cancer tissues cathepsin H was detected as three protein bands measuring approximately 31, 29 and 22 kDa (Figure 5). The frequency and levels of expression for these cathepsin H protein forms differed between normal mucosa and cancer tissues.

### Expression of the 31/29 kDa cathepsin H protein doublet

The most typical cathepsin H banding pattern for control mucosa was a protein doublet with bands measuring approximately 31 and 29 kDa (Figure 5A). This doublet was significantly more common in normal mucosae (41/43) than in corresponding cancer specimens (28/43) ($P$ = 0.001). A quantitative change occurred in the cancers, with decreased levels of the 31 kDa cathepsin H band detected in 90% of the 43 cancers studied compared to the matched normal mucosa. In some cancers the 31 kDa cathepsin H band was undetectable, even when films of the Western blot data were overexposed. For 28 cases that expressed the 31 kDa band at detectable levels in both cancer and matched normal samples, the C/N ratio for this form of cathepsin H protein was 0.47 ± 0.49. In contrast to the loss of expression of the 31 kDa band in cancers, most cancers demonstrated the clear presence of a 29 kDa mature single-chain form of cathepsin H at levels that were usually unchanged or increased compared to the normal mucosa.

### Expression of the 22 kDa cathepsin H heavy-chain band

Up-regulation of a 22 kDa protein band was also observed in cancers versus matched mucosa samples detected on Western blots. This cathepsin H form is classically described as the 22 kDa heavy-chain of mature, active two-chain cathepsin H (the corresponding 6 kDa light-chain of this form is not detected due to gel running conditions), derived from the 29 kDa single-chain form by further processing. This 22 kDa cathepsin H protein (Figure 5B), was detected in 72% of the cancer tissues (31/43) compared to 53% of control mucosa samples (23/43 tissues). For those 23 cases that demonstrated this band in both matched cancer and normal mucosa specimens, expression levels in the normal mucosa were often very weak, with a resulting C/N of 2.9 ± 1.5 ($P$ < 0.0001). A comparison of the typical normal versus cancer pattern for the 22 kDa heavy-chain band of cathepsin H can be seen in Figure 5B. C/N ratios for the 23 cases in which this protein form was present in both control mucosa and cancers, were found to be highest in Dukes' B (3.1 ± 1.6, $n$ = 10) and Dukes' C (3.2 ± 1.8, $n$ = 7) compared to Dukes' A or D stage cancers (2.1 ± 1.0, $n$ = 3 and 1.8 ± 0.5, $n$ = 3 respectively), with C/N ratios for stages B and C significantly different from 1.0 ($P$ < 0.005 and $P$ < 0.02 respectively).

### Cathepsin H: controls for protein banding studies

As seen in Figure 5C, the specificity of the cathepsin H antibody was confirmed by detection of purified human liver cathepsin H protein but not of purified human liver cathepsins B, L or D proteins that were run side by side and probed simultaneously, on a single Western blot, with the cathepsin H antibody.

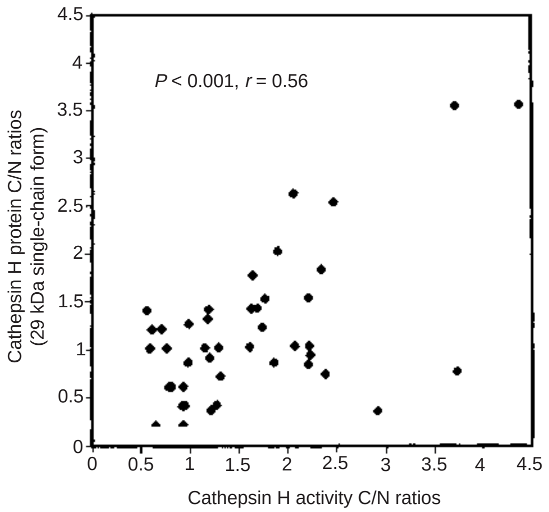

**Figure 6** Scatter plot correlation analysis of C/N ratios for cathepsin H enzyme-specific activity versus C/N ratios for the cathepsin H 29 kDa mature protein form. The C/N ratios for cathepsin H activity correlated with the C/N ratios for the 29 kDa mature cathepsin H protein form with each assay done on the identical extracts from 43 cancer/normal tissue pairs. The slope for this comparison was significantly different from 0, with $r$ = 0.56 and $P$ < 0.001

We also determined whether the alterations in cathepsin H banding patterns in cancer samples might be due to increased proteolytic processing of cathepsin H occurring in cancer homogenates following extraction. However, identical cathepsin H banding patterns were detected by Western blotting for five independent extractions of the same cancer tissue, whether the extraction was done in distilled, deionized water or in the presence of any of four inhibitors for different classes of proteolytic enzymes (data not shown).

### Cathepsin H: correlation of single-chain mature form with enzyme activity

To test for a correlation between changes in cathepsin H activity levels and cathepsin H protein levels in cancer samples compared to normal mucosa, C/N ratios for cathepsin H activity were compared to C/N ratios for cathepsin H protein levels as detected in individual cathepsin H protein bands on Western blots of 43 matched extract pairs. These comparisons of activity C/N ratios with protein C/N ratios for individual cases revealed that the common decrease in the 31 kDa band, although a very good marker for cancer, did not correlate with cathepsin H aminopeptidase activity levels. However, as seen in Figure 6, a significant correlation was observed between C/N ratios for cathepsin H activity and the corresponding C/N ratios for the 29 kDa mature single-chain form of cathepsin H in these same cases ($r$ = 0.56; $P$ < 0.001). Typically, cases with high cathepsin H activity C/N ratios (C/N ≥ 1.5) also demonstrated significantly increased C/N ratios for the 29 kDa cathepsin H protein band. However, there were exceptions to this rule, as the percentage of cases demonstrating a C/N ≥ 1.5 for the 29 kDa protein band (21% of cases analysed) was less frequent than the percentage of cases demonstrating an activity C/N ratio ≥ 1.5 (36%). For cases with high activity C/N ratios but without a marked increase in the 29 kDa band, the majority of cases (8/11) demonstrated a high C/N ratio for the 22 kDa cathepsin H protein form, suggesting that this more

© 2000 Cancer Research Campaign

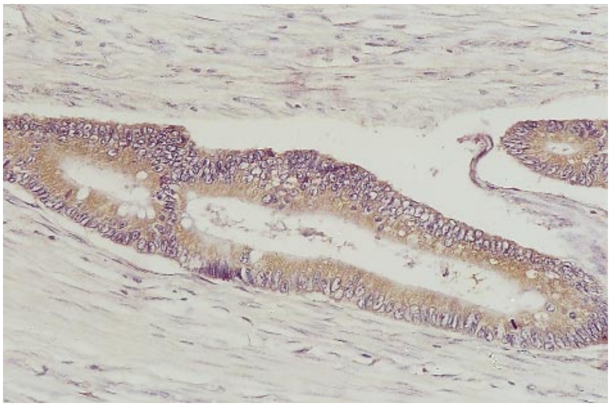

**Figure 7** Cathepsin H immunohistochemical staining in colorectal cancer. Cathepsin H protein expression in a Dukes' C stage cancer is shown, as detected on a paraffin-embedded section incubated with a 1:2500 dilution of anti-human cathepsin H antibody and staining developed using a biotin-streptavidin label, followed by a haematoxylin counterstain. This case demonstrates staining of the cancer epithelium, with intensity of staining scored as +3 to +4, while stroma surrounding the tumour epithelium was essentially negative for Cathepsin H staining. Cathepsin H protein is seen to be located diffusely throughout the cytoplasm in the form of fine granules fully processed form of cathepsin H also contributes to cathepsin H aminopeptidase activity, as previously reported (Nishimura et al, 1995).

### Cathepsin H protein: correlation with site

The sample of cases studied for cathepsin H protein expression patterns included 20 right-sided cancers and 23 left-sided cancers. The mean C/N ratio for the 29 kDa cathepsin H mature protein in left-sided cancers ($1.4 \pm 0.9$) was also found to be significantly higher than the mean C/N ratio for the 29 kDa cathepsin H protein in right-sided cancers ($0.9 \pm 0.5$) ($P < 0.05$).

### Cathepsin H immunohistochemistry

Cathepsin H protein content and cellular localization were analysed by immunohistochemical staining of tissue sections for seven colorectal cancer cases. For both normal colon mucosa and colorectal carcinoma sections, cathepsin H protein was primarily localized to the epithelial cells, although staining of macrophages was also seen. Cathepsin H staining in normal mucosa showed a punctate pattern typical of lysosomal localization, while carcinomas demonstrated a fine granular, more diffuse cytoplasmic staining. Cathepsin H protein staining was elevated in cancer epithelial cells when compared to control mucosa (present on slides for five of seven cases analysed) or to the stroma, particularly for those cases with high C/N ratios for cathepsin H activity. However, comparative statistical analyses of immunohistochemical data versus activity data on the same cases will require much larger sample sizes. Immunohistochemical staining for cathepsin H in a Dukes' C stage cancer is shown in Figure 7. This case, that had a C/N ratio of 2.9 for cathepsin H activity, demonstrated staining of the cancer epithelium, with intensity of staining scored as +3 to +4, while stroma surrounding the tumour epithelium was essentially negative for cathepsin H staining, except for macrophages (not seen in this Figure).

### DISCUSSION

Studies of melanomas, squamous cell carcinomas, gliomas, breast and pancreatic carcinomas have shown altered expression of cathepsin H by immunohistochemistry or enzyme-linked immunosorbent assays (ELISA) (Gabrijelcic et al, 1992; Budinha et al, 1996; Paciucci et al, 1996; Sivaparvathi et al, 1996; Kos et al, 1997). However, few studies have analysed cathepsin H expression in a large set of matched pairs of colorectal cancers and mucosa by activity assays or Western blots.

Our study has demonstrated significant increases in cathepsin H activity levels in colorectal cancer, alterations in cathepsin H protein banding patterns, as well as localization of the protein in the tumour epithelial cells. Particularly significant increases in cathepsin H expression in Dukes' B and C stage carcinomas suggest a correlation between elevated cathepsin H activity and the processes of local or lymph node invasion by colorectal cancer. Increases in cathepsin H protein content in melanomas (Kos et al, 1997) and in enzyme activity and protein content in gliomas (Sivaparvathi et al, 1996) also correlated significantly to the malignant potential of these cancers.

Our cathepsin H activity data also indicate that two different populations of colorectal cancers may exist, with one-third of all cases characterized by high cathepsin H C/N ratios (C/N ratios $\geq 1.5$) and the remaining two-thirds having no significant change in cathepsin H activity levels (C/N ratios $\simeq 1$). Although one previous study of five colorectal cancers and matched mucosa homogenates showed increased cathepsin H enzyme activity in the cancer extracts, the results were not statistically significant, possibly due to sample size (Keppler et al, 1988). Different subsets of colorectal cancers with very different cathepsin H expression levels may also exist, due perhaps to a site-specific role for cathepsin H in colorectal cancers, as we have observed that activities were highest and most frequently elevated in cancers from the left portion of the large intestine.

Marked differences exist between cancers of the left and right colon. Right-sided cancers are preferentially fungating, protruding into the lumen as cauliflower-like masses, while left-sided cancers tend to directly penetrate into the bowel wall (Rubin and Farber, 1994). Despite being more easily visualized by colonoscopy than cancers from the right colon, ulcerating tumours of the distal left colon are often diagnosed at a later stage and characterized by worse prognoses (Wolmark et al, 1984; Rubin and Farber, 1994). Left-sided tumours have significantly higher p53 overexpression or mutation rates (71.4–67%) than right-sided tumours (42.1–22%) (Breivnik et al, 1997; Lenz et al, 1998), while microsatellite instability (MIN) is almost exclusively associated with cancers of the proximal (right) colon (Breivnik et al, 1994, 1997). Furthermore, K-*ras* gene mutations, present in 40–60% of colorectal carcinomas, are negatively associated with the presence of MIN (Breivnik et al, 1994). Thus, it is possible to hypothesize that either K-*ras* mutations or high expression of the tumour suppressor gene p53 might correlate with increased activity of the cysteine proteinase cathepsin H in the left colon. Variations in cathepsin H expression with cancer site may reflect the different genetic pathways described for colorectal cancers in left versus right colon. Correlations between these molecular markers and cathepsin B and L activities in colorectal cancers have already been reported (Iacobuzio-Donahue et al, 1996; Kim et al, 1998).

A comparison of our cathepsin H activity data for clinical stage and site confirmed that both variables could be related to elevated

*© 2000 Cancer Research Campaign*

cathepsin H C/N ratios. Irrespective of site, C/N ratios were increased most frequently in colorectal cancers invading the bowel wall and spreading to the lymph nodes (Dukes' B and C cancers), although the more aggressive left-sided colon cancers had the most marked expression of cathepsin H. Notably, a higher frequency of node-positive cancers is also found for cancers of the left colon (Wolmark et al, 1984).

Our research has also provided new information on changes in cathepsin H protein forms in cancer compared to normal tissue. Traditionally three cathepsin H forms are described, including a 41 kDa proform, a 28 kDa mature single-chain form and a two-chain mature form consisting of the 22 kDa heavy-chain plus a 6 kDa light-chain of cathepsin H (Kirschke et al, 1998). In addition, unusual cathepsin H molecular forms have been reported for a cancer cell line (Waguri et al, 1995) and for polycystic kidney disease (Hartz and Wilson, 1997). Our Western blot data on cathepsin H protein bands has revealed a cathepsin H protein doublet at 31 and 29 kDa, present in 41/43 (95%) normal colon mucosa samples. A comparable cathepsin H protein doublet was detected on Western blots of normal and malignant brain tissues (Sivaparvathi et al, 1996). Our data indicate that reproducible changes in the expression of this protein doublet provide the most consistent indication of altered cathepsin H expression in colorectal cancers. Loss or down-regulation of the 31 kDa cathepsin H protein band occurred in 90% of the cancers analysed, irrespective of cancer stage or site. Since this decrease in the 31 kDa band in cancers is much more common than the significant increase in cathepsin H activity measured in 1/3 of the cancers, it is not yet clear how the 31 kDa band contributes to normal function nor how cathepsin H function changes with the loss of this band in cancers.

Conceivably, the 31 kDa cathepsin H protein band may represent a protein intermediate that is converted to the 29 kDa form during processing of cathepsin H zymogen. In fact, a transient cathepsin H of 30 kDa, present during the generation of a mature 28 kDa single-chain cathepsin H has been described (Nishimura and Kato, 1987). A higher rate of cathepsin H processing by proteolytic clipping in colon cancer cells may explain the consistent down-regulation of the 31 kDa band in cancers as compared to the matched mucosae. Our control extractions in the presence of different proteinase inhibitors indicated that any such processing events would have to occur in the cancer prior to extraction rather than during or after the extraction process.

For that subset of colorectal cancers, particularly Dukes' B and C stage cancers of the left colon, in which there was a significant increase in cathepsin H activity, the activity increase correlated with increased amounts of the 29 kDa single-chain cathepsin H protein form or alternatively, with increased expression of the 22 kDa heavy chain of two-chain mature cathepsin H. These data suggest again that the cathepsin H aminopeptidase activity measured in our enzyme assay are dependent on expression levels of more fully processed cathepsin H forms, including the 29 kDa mature single-chain and the mature two-chain form, represented by the 22 kDa band, rather than on the 31 kDa cathepsin H protein. Nishimura and colleagues (1995) have reported a particularly marked relationship between the presence of the 22 kDa cathepsin H band and higher cathepsin H enzyme-specific activity as measured in two distinct pools of cathepsin H protein isolated from rat liver lysosomes.

Changes in cathepsin H expression were also shown by immunohistochemical assays to be associated with increased cathepsin H protein staining in carcinoma compared to normal mucosa, with increased protein content detected primarily in cancer epithelial cells, rather than stromal cells. A shift in cathepsin H staining from a more punctate pattern in normal mucosa, typical of lysosomal localization, to a fine granular, more diffuse cytoplasmic staining in carcinomas was also observed, similar to shifts in localization reported for cathepsin B protein staining of colorectal cancers (Iacobuzio-Donahue et al, 1997).

Our results on cathepsin H expression in colorectal cancers indicate both similarities and differences to the cancer-related changes observed for cathepsins B and L in these same colorectal cancer cases (Sheahan et al, 1989; Shuja et al, 1991; Iacobuzio-Donahue et al, 1997). Cathepsin B and L activities were most significantly and frequently elevated in Dukes' A and B cancers in contrast to cathepsin H activity, which was most elevated in Dukes' B and C stage cancers. Western blot studies of protein patterns for cathepsin B (Iacobuzio-Donahue et al, 1997) and cathepsin H (this study) have supported activity analyses but have also revealed reproducible changes for both markers in the protein forms expressed in cancers versus normal mucosa. For both cathepsin B and cathepsin H, a specific change in protein banding pattern was the most consistent indication of malignancy, irrespective of stage, while changes in activity or protein expression levels were more specifically related to stages of cancer progression.

Although Dukes' classification remains a powerful predictor of final outcome for colorectal cancer patients (Dukes, 1932; Deans et al, 1992), the traditional clinicopathological predictors are not yet fully successful at predicting recurrence and survival risks for patients with Dukes' stage B and C cancers (Liefers et al, 1998). Our data on cathepsin H suggest that this new proteinase marker might be particularly useful in defining Dukes' B and C stage cancers and in distinguishing subsets of cancers at a given site. Thus, we are currently analysing cathepsin H expression with respect to patient prognoses.

### ACKNOWLEDGEMENTS

We thank pathologists and residents at the Mallory Institute of Pathology and the Cooperative Human Tissue Network (CHTN) for assistance in providing primary tissue samples for analysis. This work was supported by a grant from the ACS-MA Div., Inc. (to MJM) and by National Institute of Health Grant CA51865 (to MJM).

### REFERENCES

Barrett AJ, Kembhavi AA, Brown MA, Kirschke CG, Tamai M and Hanada K (1982) L-trans-epoxysuccinyl-leucylamido (4-guanidino)butane (E-64) and its analogues as inhibitors of cysteine proteinases including cathepsins B, H and L. *Biochem J* **201**: 189–198

Breivnik J, Lothe RA, Meling GI, Rognum TO, Borresen-Dale A and Gaudernack G (1997) Different genetic pathways to proximal and distal colorectal cancer influenced by sex-related factors. *Int J Cancer* **74**: 664–669

Breivnik J, Meling GI, Spurkland A, Rognum TO and Gaudernack G (1994) K-ras mutation in colorectal cancer: relations to patient age, sex and tumour location. *Br J Cancer* **69**: 367–371

Budinha M, Strojan P, Smid L, Skrk J, Vrhovec I, Zupevc A, Rudolf Z, Zargi M, Krasovec M, Svetic B, Kopitar-Jerala N and Kos J (1996) Prognostic value of cathepsins B, H, L, D and their endogenous inhibitors stefins A and B in head and neck carcinoma. *Biol Chem Hoppe-Seyler* **377**: 385–390

Chapman HA, Riese RJ and Shi GP (1997) Emerging roles for cysteine proteases in human biology. *Ann Rev Physiol* **59**: 63–88

© 2000 Cancer Research Campaign 

Claus V, Jahraus A, Tjelle T, Berg T, Kirschke H, Faulstich H and Griffiths G (1998) Lysosomal enzyme trafficking between phagosomes, endosomes and lysosomes in J774 macrophages. Enrichment of cathepsin H in early endosomes. *J Biol Chem* **272**: 9842–9851

Dawson-Saunders B and Trapp RG (1994) *Basic and Clinical Biostatistics*. Appleton and Lange: Norwalk, CT

Deans GT, Parks TG, Rowlands BJ and Spence RAJ (1992) Prognostic factors in colorectal cancer. *Br J Surg* **79**: 608–613

Duffy MJ (1992) The role of proteolytic enzymes in cancer invasion and metastasis. *Clin Exp Met* **10**: 145–155

Dukes CE (1932) The classification of the cancer of the rectum. *J Pathol Bacter* **35**: 323–332

Fu YF, Nishinaka T, Yokoyama K and Chiu R (1998) A retinoblastoma susceptibility gene product, RB, targeting protease is regulated through the cell cycle FEBS *Lett* **421**: 89–93

Gabrijelcic D, Svetic B, Spaic D, Skrk J, Bidinha M, Dolenc I, Popovic T, Cotic V and Turk V (1992) Cathepsins B, H and L in human breast carcinoma. *Eur J Clin Chem Clin Biochem* **30**: 69–74

Guncar G, Podobnik M, Pungercar J, Strukelj B, Turk V and Turk D (1998) Crystal structure of porcine cathepsin H determined at 2.1 Å resolution: location of the mini-chain C-terminal carboxyl group defines cathepsin H aminopeptidase function. *Structure* **6**: 51–61

Hartz PA and Wilson PD (1997) Functional defects in lysosomal enzymes in autosomal dominant polycystic kidney disease (ADPKD): abnormalities in synthesis, molecular processing, polarity and secretion. *Biochem Mol Med* **60**: 8–26

Iacobuzio-Donahue C, Cai J, Noguiera C, Shuja S, Kim K and Murnane MJ (1996) Correlation of increased cathepsin B expression with p53 loss of heterozygosity at the transition from colorectal adenoma to carcinoma. *Proc Am Ass Cancer Res* **37**: 95

Iacobuzio-Donahue CA, Shuja S, Cai J, Peng , and Murnane MJ (1997) Elevations of cathepsin B protein content and enzyme activity occur independently of glycosylation during colorectal tumor progression. *J Biol Chem* **272**: 29190–29199

Johnson GD and Hersh LB (1990) Studies on the subsite specificity of the rat brain puromycin-sensitive aminopeptidase. *Arch Biochem Biophys* **276**: 305–309

Kageshita T, Yoshii A, Kimura T, Maruo K, Ono T, Himeno M and Nishimura Y (1995) Biochemical and immunohistochemical analysis of cathepsins B, H, L and D in human melanocytic tumors. *Arch Dermatol Res* **287**: 266–272

Katunuma N and Kominami E (1995) Structure, properties, mechanisms, and assays of cysteine protease inhibitors: cystatins and E-64 derivatives. *Methods Enzymol* **251**: 382–397

Keppler D, Fondaneche MC, Dalet-Fumaron V, Pagano M and Burtin P (1988) Immunohistochemical and biochemical study of a cathepsin B proteinase in human colonic cancers. *Cancer Res* **48**: 6855–6862

Kim K, Cai J, Shuja S, Kuo T and Murnane MJ (1998) Presence of activated ras correlates with increased cysteine proteinase activities in human colorectal carcinomas. *Int J Cancer* **79**: 324–333

Kirschke H, Barrett AJ and Rawlings ND (1998) *Lysosomal Cysteine Proteinases.* Oxford University Press: New York

Kos J, Smid A, Krasovec M, Svetic B, Lenarcic B, Vrhovec I, Skrk J and Turk V (1995) Lysosomal proteases cathepsins D, B, H, L and their inhibitors stefins A and B in head and neck cancer. *Biol Chem Hoppe-Seyler* **376**: 401–405

Kos J, Stabuc B, Schweiger A, Krasovec M, Cimerman N, Kopitar-Jerala N and Vrhovec I (1997) Cathepsins B, H and L and their inhibitors stefin A and cystatin C in sera of melanoma patients. *Clin Cancer Res* **3**: 1815–1822

Lafuse WP, Brown D, Castle L and Zwilling BS (1995) IFN-gamma increases cathepsin H mRNA levels in mouse macrophages. *J Leukoc Biol* **57**: 663–669

Lenz HJ, Danenberg KD, Leichman CG, Florentine B, Johnston PG, Groshen S, Zhou L, Xiong YP, Danenberg PV and Leichman LP (1998) p53 and thymidilate synthase expression in untreated stage II colon cancer: associations with recurrence, survival, and site. *Clin Cancer Res* **4**: 1277–1234

Leto G, Tumminiello FM, Pizzolanti G, Montalto G, Soresi M, Carroccio A, Ippolito S and Gebbia N (1997) Lysosomal aspartic and cysteine proteinases serum levels in patients with pancreatic cancer or pancreatitis. *Pancreas* **14**: 22–27

Liefers GJ, Cleton-Jansen AM, van de Velde CJH, Hermans J, van Krieken JHJM, Cornelisse CJ and Tollenaar AEM (1998) Micrometastases and survival in stage II colorectal cancer. *N Engl J Med* **339**: 223–228

Liotta LA and Stetler-Stevenson WG (1991) Tumor invasion and metastasis: an imbalance of positive and negative regulation. *Cancer Res* **51**: 5054–5059

Lowry O, Rosebrough NJ, Farr AL and Randall RJ (1951) Protein measurements with the folin phenol reagent. *J Biol Chem* **193**: 265–275

Mathur PP, Grima J, Mo MY, Zhu LJ, Aravindan GR, Calcagno K, O'Bryan M, Chung S, Mruk D, Lee WM, Silvestrini B and Cheng CY (1997) Differential expression of multiple cathepsin mRNAs in the rat testis during maturation and following lonidamine induced tissue restructuring. *Biochem Molec Biol Intern* **42**: 217–233

Mithofer K, Fernandez-del Castillo C, Rattner D and Warshaw AL (1998) Subcellular kinetics of early trypsinogen activation in acute rodent pancreatitis. *Am J Physiol* **274**: G71–79

Nishimura Y and Kato K (1987) Identification of latent procathepsin H in microsomal lumen: characterization of proteolytic processing and enzyme activation. *Arch Biochem Biophys* **260**: 712–718

Nishimura Y, Tsuji H, Sato H, Amano J and Himeno M (1995) Biochemical properties and intracellular processing of lysosomal cathepsin B and H. *Biol Pharm Bull* **18**: 829–836

Nitatori T, Sato N, Kominami E and Uchiyama Y (1996) Participation of cathepsins B, H and L in perikaryal condensation of CA1 pyramidal neurons undergoing apoptosis after brief ischemia. In: *Intracellular Protein Catabolism*, Suzuki K and Bond J (eds), pp. 177–185. Plenum Press: New York

Paciucci R, Berrozpe G, Tora' M, Navarro E, Garcia de Herreros A and Real FX (1996) Isolation of tissue-type plasminogen activator, cathepsin H, and non-specific cross-reacting antigen from SK-PC-1 pancreas cancer cells using subtractive hybridization. *FEBS Lett* **385**: 72–76

Rubin E and Farber JL (1994) *Pathology*, JB Lippincott: Philadelphia

Schwartz MK (1995) Tissue cathepsins as tumor markers. *Clin Chim Acta* **237**: 67–78

Schwartz WN and Barrett AJ (1980) Human cathepsin *H Biochem J* **191**: 487–497

Sheahan K, Shuja S and Murnane MJ (1989) Cysteine protease activities and tumor development in human colorectal carcinomas. *Cancer Res* **49**: 3809–3814

Shuja S, Sheahan K and Murnane MJ (1991) Cysteine endopeptidase activity levels in normal human tissues, colorectal adenomas and carcinomas. *Int J Cancer* **49**: 341–346

Sivaparvathi M, Sawaya R, Gokaslan ZL, Rao JS and Chintala SK (1996) Expression and the role of cathepsin H in human glioma progression and invasion. *Cancer Lett* **107**: 161–167

Taniguchi K, Tomita M, Kominami E and Uchiyama Y (1993) Cysteine proteinases in rat dorsal root ganglion and spinal cord, with special reference to the co-localization of these enzymes with calcitonin gene-related peptide in lysosomes. *Brain Res* **601**: 143–153

Towbin H, Staehelin J and Gordon J (1979) Electrophoretic transfer of proteins from polyacrylamide gels to nitrocellulose sheets: procedure and some applications. *Proc Natl Acad Sci USA* **76**: 4350–4353

Tsushima H, Ueki A, Matsuoka Y, Mihara H and Hopsu-Havu V (1991) Characterization of a cathepsin H-like enzyme from a melanoma cell line. *Int J Cancer* **48**: 726–732

Turk B, Turk V and Turk D (1997) Structural and functional aspects of papain-like cysteine proteinases and their protein inhibitors. *Biol Chem* **378**: 141–150

Turnbull RB, Kyle K, Watson FR and Spratt J (1967) Cancer of the colon: the influence of the no-touch isolation technique on survival rates. *Ann Surg* **166**: 420–427

Waguri S, Sato N, Wanatabe T, Ishidoh K, Kominami E, Sato K, and Uchiyama Y (1995) Cysteine proteinases in GH4C1 cells, a rat pituitary tumour cell line, are secreted by the constitutive and regulated secretory pathways. *Eur J Cell Biol* **67**: 308–318

Wolmark N, Fisher ER, Wienand HS, Fisher B (1984) The relationship of depth of penetration and tumor size to the number of positive nodes in Dukes' C colorectal cancer. *Cancer* **53**: 2707–2712

 *© 2000 Cancer Research Campaign*
